# Iron Oxide Nanoparticle-Micelles (ION-Micelles) for Sensitive (Molecular) Magnetic Particle Imaging and Magnetic Resonance Imaging

**DOI:** 10.1371/journal.pone.0057335

**Published:** 2013-02-20

**Authors:** Lucas W. E. Starmans, Dirk Burdinski, Nicole P. M. Haex, Rik P. M. Moonen, Gustav J. Strijkers, Klaas Nicolay, Holger Grüll

**Affiliations:** 1 Department of Biomedical Engineering, Eindhoven University of Technology, Eindhoven, The Netherlands; 2 Department of Minimally Invasive Healthcare, Philips Research Eindhoven, Eindhoven, The Netherlands; Aristotle University of Thessaloniki, Greece

## Abstract

**Background:**

Iron oxide nanoparticles (IONs) are a promising nanoplatform for contrast-enhanced MRI. Recently, magnetic particle imaging (MPI) was introduced as a new imaging modality, which is able to directly visualize magnetic particles and could serve as a more sensitive and quantitative alternative to MRI. However, MPI requires magnetic particles with specific magnetic properties for optimal use. Current commercially available iron oxide formulations perform suboptimal in MPI, which is triggering research into optimized synthesis strategies. Most synthesis procedures aim at size control of iron oxide nanoparticles rather than control over the magnetic properties. In this study, we report on the synthesis, characterization and application of a novel ION platform for sensitive MPI and MRI.

**Methods and Results:**

IONs were synthesized using a thermal-decomposition method and subsequently phase-transferred by encapsulation into lipidic micelles (ION-Micelles). Next, the material and magnetic properties of the ION-Micelles were analyzed. Most notably, vibrating sample magnetometry measurements showed that the effective magnetic core size of the IONs is 16 nm. In addition, magnetic particle spectrometry (MPS) measurements were performed. MPS is essentially zero-dimensional MPI and therefore allows to probe the potential of iron oxide formulations for MPI. ION-Micelles induced up to 200 times higher signal in MPS measurements than commercially available iron oxide formulations (Endorem, Resovist and Sinerem) and thus likely allow for significantly more sensitive MPI. In addition, the potential of the ION-Micelle platform for molecular MPI and MRI was showcased by MPS and MRI measurements of fibrin-binding peptide functionalized ION-Micelles (FibPep-ION-Micelles) bound to blood clots.

**Conclusions:**

The presented data underlines the potential of the ION-Micelle nanoplatform for sensitive (molecular) MPI and warrants further investigation of the FibPep-ION-Micelle platform for *in vivo*, non-invasive imaging of fibrin in preclinical disease models of thrombus-related pathologies and atherosclerosis.

## Introduction

Cancer is one of the leading causes of death in the western world with a still increasing prevalence due to the aging society. As survival rates strongly increase with earlier diagnosis, much effort is devoted to improve the sensitivity and specificity of imaging methods such as PET, SPECT, CT, and MRI to detect smaller lesions. MRI offers superb contrast for soft tissue at high resolution and is often the modality of choice to characterize malignant tissue. However, MRI suffers from low sensitivity, which triggered the development of disease specific contrast agents. Especially iron oxide nanoparticles (IONs) have been investigated extensively as contrast agents for magnetic resonance imaging (MRI) during the past few decades [1], [2], [3], [4], [5]. Commonly referred to as negative contrast agents, IONs predominantly generate signal voids in MR images due to their high transversal relaxivity. They generally display good biocompatibility profiles, lack non-endogenous elements (unlike Gd^3+^-based MR contrast agents), contain a high payload per nanoparticle and can be functionalized with binding molecules, such as antibodies and peptides, which are typically linked covalently to their surface. Therefore, IONs are a promising and frequently employed nanoplatform for molecular and cellular MR imaging [6], [7], [8], [9], [10], [11], [12], [13]. A drawback of ION-based contrast-enhanced MRI is that it is often necessary to acquire both pre- and post-contrast enhanced images, which could lead to problems with respect to image co-registration and patient compliance. In addition, negative contrast ION-MRI is relatively prone to yield ambiguous information as signal voids, which may also be caused by imaging artifacts, might disguise morphology in the area of interest.

Recently, magnetic particle imaging (MPI) was introduced as a new imaging modality that is able to directly visualize magnetic particles rather than their effect on proton relaxation that is the basis of MRI detection [14]. In MPI, IONs are magnetized by a radiofrequency magnetic field and their time-varying, non-linear magnetization induces a time-dependent voltage in a receiver coil. The non-linearity of the magnetization response induces higher harmonics, the intensity of which directly corresponds to the respective ION concentration. This technique allows for radiation-free, hotspot-like imaging and should provide a more sensitive and quantitative alternative for molecular and cellular MRI [15]. A promising preclinical *in vivo* proof of concept MPI study using Resovist has already been reported [16]. Besides considerable efforts to improve MPI scanner design and image processing, optimization of IONs for MPI is critical for applications of MPI beyond proof-of-principal preclinical studies. Although it was immediately recognized by the inventers of MPI that the properties of the IONs are crucial for MPI [14], relatively limited effort has been devoted to the synthesis of iron oxide nanoparticles for MPI purposes [17], [18], [19]. Commercially available contrast agents that are currently being employed for MPI studies are not optimized for MPI and thus prevent current MPI studies from reaching optimal resolution and sensitivity. For example, only 3% of the particles in Resovist, a commercially available contrast agent used in most MPI studies, have an appropriate particle diameter to be able to contribute significantly to the MPI signal [14]. Resovist contains iron oxide nanoparticles with an average nanocrystal size of 4–6 nm [20], [21], whereas larger-sized iron oxide nanocrystal cores that display steeper magnetization curves would be more beneficial for MPI purposes. Simulation studies suggested that using monodisperse iron oxide nanocrystal cores with a size of 30 nm could increase the obtained MPS signal by a factor of about 30 [14].

**Figure 1:**
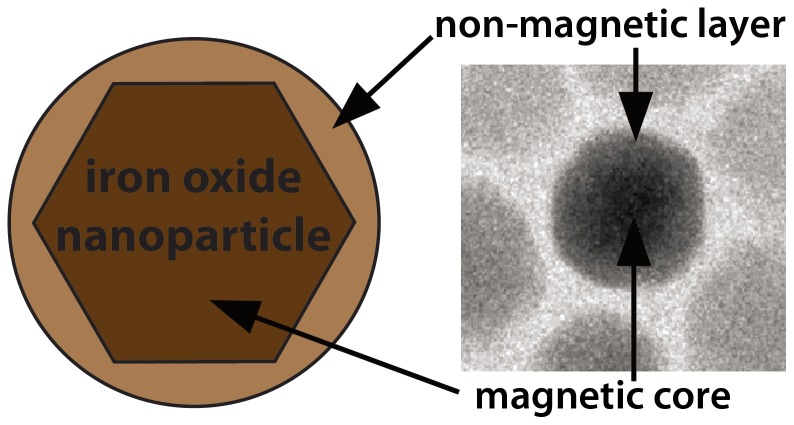
Schematic overview and TEM-micrograph of an ION displaying a magnetic core and a non-magnetic layer.

Several different procedures were published in the recent years that allow a size controlled synthesis of particles in the size range between 5–25 nm, often based on a thermal decomposition of an iron-based precursor [22], [23], [24]. Usually, particles are obtained with a magnetically active crystalline core surrounded by an amorphous layer of iron oxides (Figure 1). An analysis of the magnetic properties reveals that most particles show a smaller size of the magnetic active core with a high polydispersity compared to the geometrical size obtained e.g. with TEM [25]. Consequently, these particles will likely show a poor performance in MPI. Hence, synthesis procedures should be optimized to yield iron oxide nanoparticles with a well defined crystal core-size large enough to allow for more sensitive MPI. Naturally, these particles will also show improved magnetic properties in MR imaging.

In this study, we developed a novel iron oxide nanoparticle-micelle platform (ION-Micelle), containing iron oxide nanocrystals cores of 25 nm, for (molecular) MPI and MRI purposes. The synthesized ION-Micelles were characterized using (cryo) transmission electron microscopy (cryo-TEM), dynamic light scattering (DLS) and vibrating sample magnetometry (VSM). The effective magnetic core size of the ION-Micelles was derived from the VSM measurements. In addition, their potency for MPI and MRI was assessed using magnetic particle spectrometry (MPS) [26] and proton relaxometry, respectively. Furthermore, as a showcase for the suitability of the presented ION-Micelle platform for molecular MPI and MRI, the ION-Micelles were functionalized with a fibrin-binding peptide (FibPep) and *in vitro* blood-clot binding was assessed using MPS and MRI.

## Materials and Methods

### Materials

All materials were purchased from Sigma-Aldrich unless otherwise specified. 1,2-distearoyl-*sn*-glycero-3-phosphoethanolamine-N-[methoxy(polyethyleneglycol)-2000] (PEG2000–DSPE) was obtained from Lipoid and 1,2-distearoyl-sn-glycero-3-phosphoethanolamine-N-[maleimide(polyethyleneglycol)-2000] (Mal–PEG2000–DSPE) was obtained from Avanti Polar Lipids. 9-fluorenylmethyloxycarbonyl–protected amino acids and Rink amide resin were purchased from either Novabiochem(Merck) or Bachem and succinimidyl acetylthioacetate (SATA) was obtained from Invitrogen. Human tissue factor was purchased from Dade Behring and citrated human blood plasma was obtained from Sanquin. Resovist was purchased from Bayer Schering Pharma and Endorem and Sinerem were obtained from Guerbet.

### Iron oxide nanoparticle (ION) synthesis

IONs were obtained through a method based on thermal decomposition of FeO(OH) in the presence of oleic acid, which was developed by Yu and coworkers [22] and subsequently optimized for our purposes [27]. In short, FeO(OH) (0.10 g, 1.13 mmol) was grounded using mortar and pestle and subsequently mixed with oleic acid (3.80 g, 13.5 mmol) and eicosane (0.60 g, 2.12 mmol). The mixture was heated to 360 °C under vigorously magnetic stirring at a constant ramping rate of 3.3 °C/min and then kept at this temperature for 2 hours. During the reaction, the color of the mixture changed from red/brownish to black. After the reaction, the sample was cooled down and 10 mL of hexane was added to the mixture. The sample was precipitated using 20 mL of acetone and centrifuged at 4671 g during 30 minutes at RT. The iron oxide particles were redispersed using 10 mL of hexane and 20 µL oleic acid. The precipitation-redispersion procedure was repeated twice.

### Iron oxide nanoparticle–micelle (ION-Micelle) synthesis

IONs were phase-transferred by encapsulation of IONs into lipidic micelles according to a similar procedure used to phase-transfer quantum dots [28], [29]. In a typical phase-transfer procedure, PEG2000-DSPE (0.20 g, 71.3 µmol) was dissolved in 4 mL chloroform and 1 mL IONs in hexane (6 mg Fe) was added. A ∼40-fold excess of lipids required to entirely cover the surface of all IONs with a lipid monolayer was used. This suspension was injected into stirred, deionized water at 80 °C with an injection-speed of 6 mL/hr. Upon evaporation of the organic solvents, the IONs were encapsulated in the core of phospholipidic micelles, creating iron oxide nanoparticles-micelles (ION-Micelles). Next, phospholipid micelles without nanocrystals were removed by ultracentrifugation (42875 g; RT; 15 min) using an Optima™ L-90K ultracentrifuge equipped with a type 70.1 TI rotor (Beckman Coulter). After ultracentrifugation, the supernatant containing the empty lipidic micelles was carefully aspirated and discarded. The pellet, containing the ION-Micelles, was redispersed in HBS (10 mM HEPES, 137 mM NaCl, pH 7.4) by gently shaking. This ultracentrifugation procedure was repeated one more time.

### Nanoparticle characterization

IONs were characterized by transmission electron microscopy (TEM) and electron diffraction using a FEI Tecnai 20, type Sphera TEM instrument operating at 200 kV. Cryogenic-TEM (cryo-TEM) of ION-Micelles was performed using a Gatan cryoholder at approximately -170 °C. Sample vitrification was carried out using an automated vitrification robot (FEI Vitrobot Mark III). The hydrodynamic size of the ION-Micelles in HBS was determined with dynamic light scattering (DLS) on a Zetasizer Nano-S (Malvern) using intensity- and number-weighted particle size distributions. In addition, the hydrodynamic particle size of three commercially available iron oxide compounds (Endorem, Resovist and Sinerem) was analyzed using DLS.

Magnetization curves were obtained from ION-Micelles in HBS using a vibrating sample magnetometer (ADE Technologies). The magnetic field was varied in the range between -20 and 20 kOe. Tubes solely filled with HBS were used to correct for the diamagnetic contribution of the solvent. Effective magnetic core sizes of the ION-Micelles were determined as described by Luigjes and coworkers [25]. In short, the magnetic dipole moment distribution of the ION-micelles was calculated by fitting the magnetization curves assuming a log-normal distribution of the magnetic dipole moments. Subsequently, a volume-weighted effective magnetic core size distribution was derived from the calculated magnetic dipole moments, assuming spherical particles and a theoretical bulk magnetization of magnetite (∼90 Am^2^/kg). The volume-weighted effective magnetic core size distribution was converted to a number-weighted size distribution to allow for a more reasonable comparison between the obtained (volume-weighted) effective magnetic core size distribution and the (number-weighted) nanoparticle size distribution derived from TEM measurements.

Magnetic particle spectrometry was performed with a dedicated magnetic spectrometer (Philips) [17], [26], which is essentially a zero-dimensional version of an MPI scanner. Signal was obtained over 30 s upon application of an oscillating magnetic field with an amplitude of 10 mT at 25 kHz. As a reference, MPS measurements were also performed using Endorem, Resovist and Sinerem. Longitudinal and transverse proton relaxivities (mM^−1^s^−1^) of the ION-Micelles were determined using a Minispec MQ60 Spectrometer (1.41 T; 37 °C; Bruker). Iron concentrations were determined using inductively coupled plasma atomic emission spectrometry (ICP-AES).

### Peptide synthesis

Peptide Ac-RWQPCPAESWT-Cha-CWDPGGGK-NH_2_, containing the fibrin binding motif RWQPCPAESWT-Cha-CWDP (previously identified via phage-display using fibrinogen-binder depleted libraries and subsequently optimized for fibrin-binding) [30], [31], and a scrambled negative control Ac-WPTAD-Cha-RAWPSQEWPAGGGK-NH_2_ with C-A substitutions were synthesized on 4-methylbenzhydrylamine hydrochloride salt (MBHA) rink amide resin by the use of standard 9-fluorenylmethyloxycarbonyl solid-phase peptide synthesis [32]. The peptides were cleaved from resin by trifluoroacetic acid: triisopropylsilane: H_2_O: ethanedithiol (90.5:5:2.5:2 v/v%) for 3 hours and purified by preparative reversed-phase high-pressure liquid chromatography (RP-HPLC) using an Agilent 1200 apparatus, equipped with a C18 Zorbax column (length x internal diameter  =  150×21.2 mm; particle size, 5.0 µm). Peptide Ac-RWQPCPAESWT-Cha-CWDPGGGK-NH2 was cyclisized (formation of a disulfide bond between both cysteine residues) by dimethylsulfoxide:H_2_O (9:1 v/v%) for 5 days with the pH set to 8 using n-methyl-d-glucamine,[31] and subsequently purified using preparative RP-HPLC. Next, the peptides were functionalized at the lysine ε-amino group with a SATA group by mixing the peptides and a 10 fold excess of SATA in dimethylformamide containing 3.6 v/v% triethylamine at room-temperature overnight. Subsequently, the peptides were purified using preparative RP-HPLC, yielding the SATA-functionalized fibrin-binding peptide (FibPep) and negative control (NCFibPep) (Figure S1). Peptide structures were analyzed by liquid chromatography–mass spectrometry (LC-MS) on an Agilent 1200 apparatus, equipped with a C8 Eclipse plus column (100×2.1 mm; particle size, 3.5 µm) and an electrospray mass spectrometer (Agilent Technologies model 6210, time-of-flight LC-MS).

### Fibrin-binding peptide functionalized ION-Micelle (FibPep-ION-Micelle) and negative control fibrin-binding peptide functionalized ION-Micelle (NCFibPep-ION-Micelle) synthesis

To create fibrin-binding FibPep-ION-Micelles, the phase-transfer procedure was slightly modified. In a typical phase-transfer procedure, PEG2000-DSPE (0.525 g, 187.1 µmol) and MAL-PEG2000-DSPE (0.061 g, 20.7 µmol) were dissolved in chloroform (4 mL) and IONs in hexane (2 ml; 12 mg Fe) were added. ION-Micelles were formed and subsequently separated from empty micelles using the previously described injection and ultracentrifugation procedure. After ultracentrifugation, the ION-Micelle pellets were redispersed in HBS (pH 6.7) and the ultracentrifugation procedure was repeated one more time. In parallel to the phase-transfer procedure, the SATA-groups of the fibrin-binding peptides were deacetylated at RT for 1 hr using a hydroxylamine containing buffer (Figure S1). Subsequently, fibrin-binding peptide functionalized ION-Micelles (FibPep-ION-Micelles) were created by coupling the deacetylated fibrin-binding peptides (200 nmol peptide) to the distal end of the MAL-PEG2000-DSPE lipids overnight at 4 °C, using half of the obtained ION-Micelle suspension. Negative control fibrin-binding peptide functionalized micelles (NCFibPep-ION-Micelles) were obtained by conjugating NCFibPep to the remaining half of the ION-Micelle suspension in a similar fashion. Next, the FibPep/NCFibPep-ION-Micelles were prepared for *in vitro* application by removing any precipitation/large aggregates using centrifugation (500 g; 5 min) and subsequently changing the buffer to HBS (pH 7.4) using centrifugation-filtration (Vivaspin 50 k MWCO, 6 ml; Sartorius Stedim Biotech).

### Blood clot assay

Blood clots were prepared by incubating a mixture of 1.3 µL human tissue factor, 1.5 µL of 1 M CaCl_2_ in ultrapure water and 100 µL of citrated human blood plasma for 30 minutes at 37 °C. Subsequently, the blood clots were washed 3x with HBS. Next, 15 µl of FibPep-ION-Micelles or NCFibPep-ION-Micelles (8 µg Fe per sample; n = 4 per group) and 30 µl HBS were added to the blood clots and incubated for 1 hour at RT. Subsequently, the solution was removed and the blood clot was washed 3x with HBS. The clots were then photographed and subjected to MPS and MRI measurements. MR imaging was performed on a 9.4 T preclinical scanner (Bruker BioSpin) using a 35 mm birdcage transceiver volume coil. Sagittal T_1_ weighted images were acquired using a FLASH sequence with the following settings: TE 3.2 ms, TR 90 ms, 40° flip angle, 6 averages, FOV 25×20 mm^2^, matrix 500×400, pixel dimensions 0.05×0.05 mm^2^ and a slice thickness of 0.5 mm. Transversal slices were obtained using a TE of 3.6 ms, FOV of 50×20 mm^2^ and a matrix of 1000×400. Signal to noise ratio of the blood clots in the transversal scans were calculated by drawing a region of interest (ROI) around each vial and subsequently dividing the mean MR signal in the ROI by 0.8 x mean MR noise level. Iron content of the clots was determined using ICP-AES.

### Statistical analysis

For differences between 2 groups, data sets were compared using unpaired 2-sided *t-*test (not assuming equal variances). Values of p<0.05 were considered significant.

## Results and Discussion

**Figure 2:**
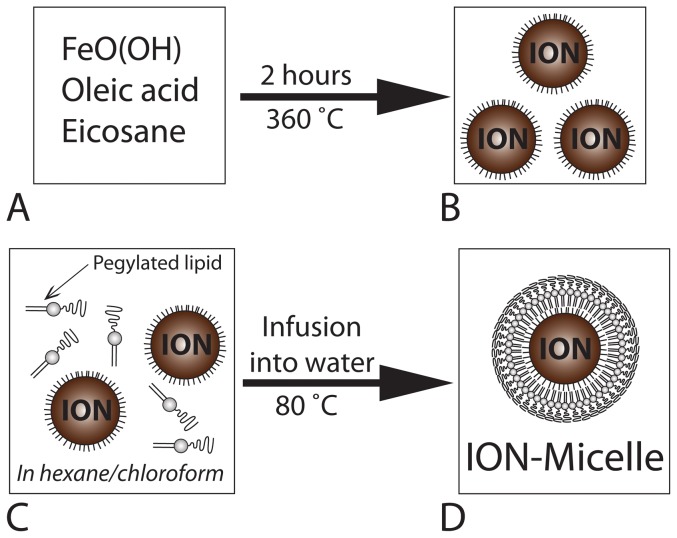
Schematic overview of the (A–B) iron oxide nanoparticles and (C–D) ION-Micelle synthesis.

**Figure 3:**
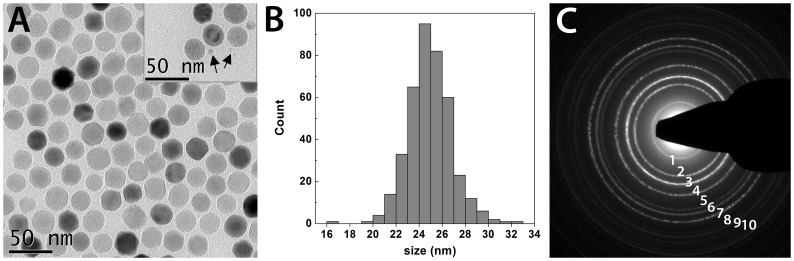
Transmission electron microscopy analysis of the IONs. (A) Typical TEM micrograph of the IONs; inset shows occasional presence of a subset of particles with a smaller diameter (arrows). (B) Size distribution profile of the IONs obtained from TEM analysis of 400 nanoparticles. (C) Selected area electron diffraction (SAED) pattern acquired from IONs.

Iron oxide nanoparticles (IONs) were synthesized by heating a mixture of FeO(OH), oleic acid and eicosane to 360 °C for 2 hours (Figure. 2A–B) as described by Burdinski and coworkers [27]. IONs were subsequently purified by precipitation and re-dispersion of the nanoparticles, using acetone and hexane respectively. Figure 3A shows a TEM image of the obtained IONs. The nanoparticles were relatively monodisperse, with an average size of 24.9 ± 1.9 nm (calculated from 400 nanoparticles; Figure 3B). Occasionally, a minor fraction of much smaller IONs was observed as well (arrows; inset Figure 3A). The diameter of the synthesized IONs was approximately a factor 5 larger compared with Endorem, Resovist and Sinerem (Table 1), which were used as benchmark iron oxide formulations in this study. Figure 3C displays a selected area electron diffraction (SAED) pattern acquired from the synthesized IONs. The measured lattice spacings based on the rings in the diffraction patterns match well with the known lattice spacings of magnetite (Table 2).

**Table 1:** Physical characteristics and relaxivities. Relaxometric measurements were performed at 1.41 T and 37 °C.

Compound	Diameter iron oxide core (nm)	Hydrodynamic diameter* (nm)	r_1_ (mM^−1^ s^−1^)	r_2_ (mM^−1^ s^−1^)	r_2_/r_1_
ION-Micelle	25	61	6.7	253	37.7
Endorem	4–6 [33]	116	8.5	80	9.4
Resovist	4–6 [20], [21]	62	9.9	119	12.0
Sinerem	4–6 [33]	32	9.5	67	7.1

r_1_: longitudinal relaxivity;

r_2_: transversal relaxivity;

* Z-average

**Table 2:** Calculated atomic lattice spacing d (Å) corresponding to diffraction pattern in Fig. 3C compared to standard atomic spacing for bulk magnetite (Fe_3_O_4_) along with their respective hkl indices from the PDF database.

d	4.87	2.95	2.51	2.07	1.70	1.60	1.47	1.31	1.27	1.20
Fe_3_O_4_	4.86	2.97	2.53	2.10	1.71	1.62	1.48	1.33	1.28	1.21
Ring	1	2	3	4	5	6	7	8	9	10
hkl index	111	220	311	400	422	511	440	620	533	444

**Figure 4:**
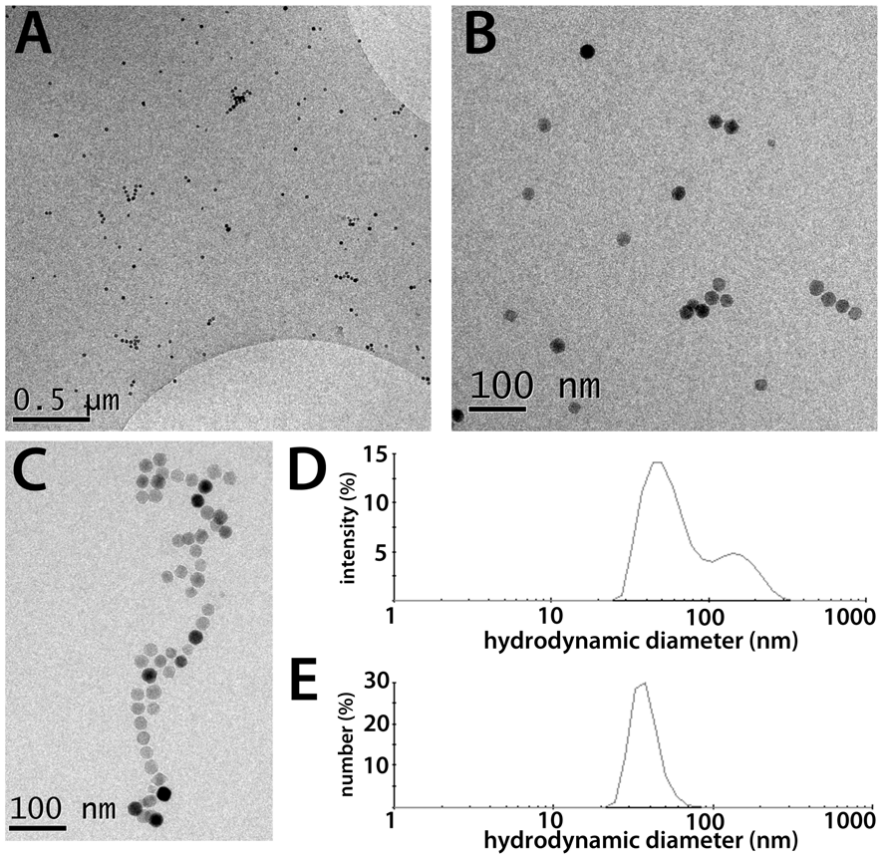
Cryo-TEM and DLS analysis of the ION-Micelles in HEPES buffered saline (HBS). (A,B) Typical Cryo-TEM micrographs of the ION-Micelles showing that the ION-Micelles are mostly dispersed as single particles or as small aggregates of particles in HBS. (C) Occasionally, larger ION-Micelle aggregates were observed. (D) Typical intensity-weighted and (E) number-weighted size-distribution profiles of the ION-Micelles obtained by dynamic light scattering measurements.

**Figure 5:**
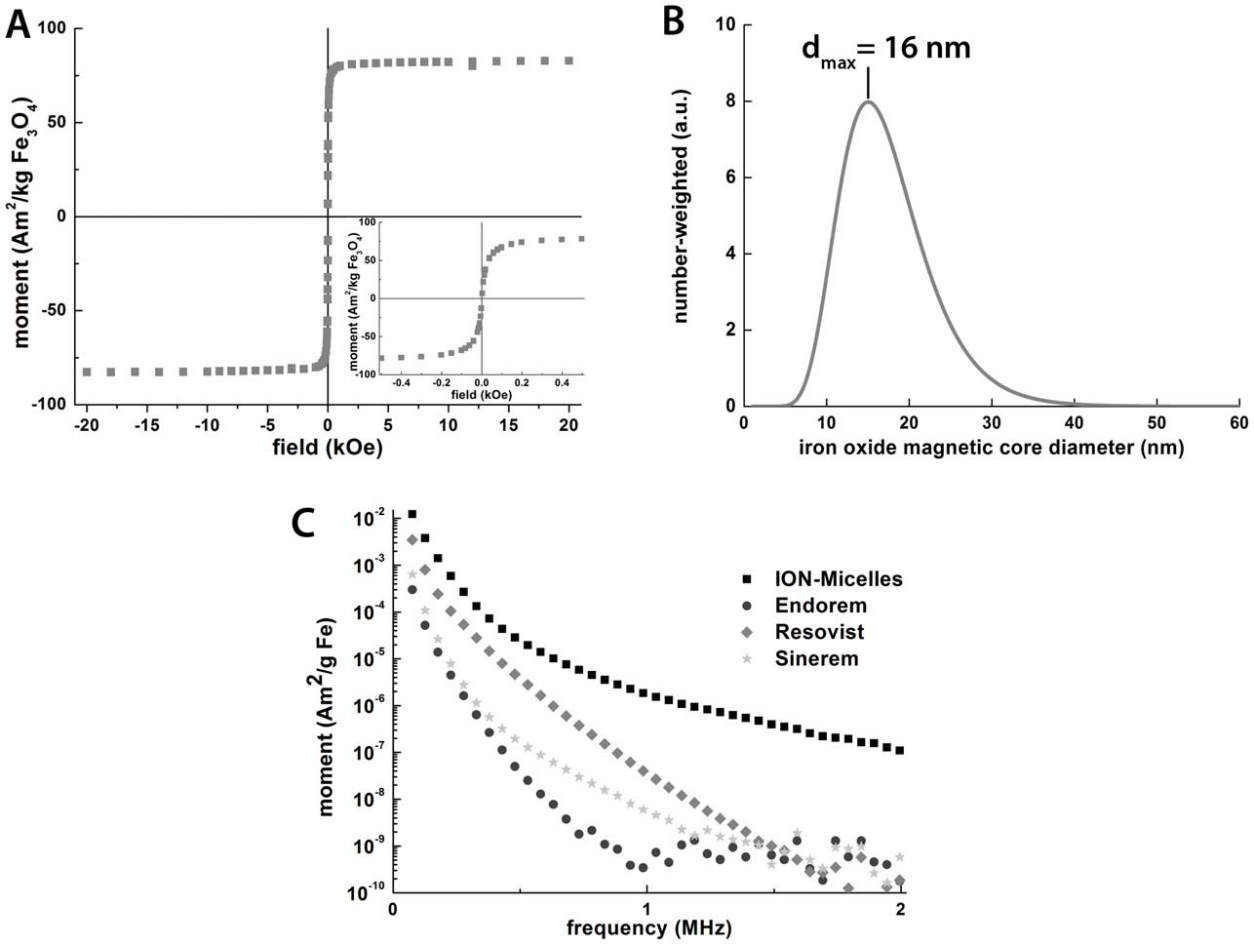
Analysis of ION-Micelles magnetic properties. (A) Magnetization curve of the ION-Micelles at room temperature. Inset: zoomed-in view around zero field. (B) Number-weighted particle size distribution of the ION-Micelles calculated from the magnetization curve. d_max_ is the diameter corresponding to the maximum of the peak. (C) MPS experimental data of the ION-Micelles, Endorem, Resovist and Sinerem plotted as magnetic moment (normalized for iron content) versus frequency.

Water-soluble ION-Micelles were formed by infusing a hexane/chloroform mixture containing IONs and 1,2-Distearoyl-*sn*-glycero-3-phosphoethanolamine-N-[methoxy(polyethyleneglycol)-2000] (PEG2000–DSPE) lipids into ultrapure water at 80 °C (Figure. 2C–D). ION-Micelles were separated from micelles not containing an iron oxide core using ultracentrifugation and subsequently the ION-Micelles were redispersed in HEPES-buffered saline (HBS) at pH 7.4. The dispersion state of the ION-Micelles in HBS was studied using cryogenic-TEM (cryo-TEM) and dynamic light scattering (DLS). Figure 4A–B shows typical high-resolution cryo-TEM images of ION-Micelles. The ION-Micelles were mostly dispersed in HBS as single particles or as small aggregates of nanoparticles. Occasionally, also larger, worm-like aggregates were observed (Figure 4C). Other lipidic structures, such as liposomes and (empty) micelles, were not observed. Figure 4D shows the ION-Micelle hydrodynamic size-distribution obtained from an intensity-weighted analysis of the time correlation function measured with DLS. One dominant peak was observed at 47 nm for the ION micelles. The minor peak at larger sizes was indicative of a small fraction of aggregated nanoparticles, which matches well with the cryo-TEM findings (Figure 4A–C). This peak disappeared in the number-weighted analysis (Figure 4E), implying that the relative contribution of the larger sized aggregates to the overall size-distribution was negligible. The hydrodynamic diameter of the ION-Micelles obtained using number-weighted analysis was 38 nm, which corresponds well with the nanoparticles core size-measurements (25 nm, Figure 3B). The apparent increase of the hydrodynamic size as determined by DLS compared to the nanoparticles core size measured by TEM is caused by the (hydrated) PEG2000-DSPE coating of the particles and the fact that a fraction of the particles contains multiple iron oxide cores. The hydrodynamic diameter of the ION-Micelles was similar to Resovist, approximately a factor two larger than Sinerem and a factor two smaller than Endorem (Table 1). The magnetic properties of the ION-Micelles were analyzed using a vibrating sample magnetometer (VSM). The ION-Micelles behaved superparamagnetically at room-temperature (RT), as the magnetization curve had no hysteresis (Figure 5A). The saturation magnetization of the ION-Micelles was found to be 82 Am^2^/kg Fe_3_O_4_, which is relatively close to the saturation magnetization of bulk magnetite (∼90 Am^2^/kg Fe_3_O_4_ at RT).

It has been recognized that the usual assumption that uniform size and shape guarantee well-defined magnetic properties is often in stark contrast with reality. For instance, Luigjes and coworkers showed that two formulations of iron oxide nanoparticles, both with a 20 nm size on TEM, displayed significantly different effective magnetic core sizes (16 and 6 nm), which were calculated from the magnetization curves [25]. As the magnetic properties of the nanoparticles are likely the sole critical factor for MPI-purposes, we calculated the effective magnetic core size distribution of our ION-Micelles as described by Luigjes et al. [25]. Figure 5B shows the obtained number-weighted magnetic core size distribution, which displayed a maximum at 16 nm. The fact that the effective magnetic core size of the ION-Micelles is already larger than the average size of an entire iron oxide core in Resovist (4–6 nm), suggests that the ION-Micelles will allow for significantly more sensitive MPI.

Magnetic particle spectrometry (MPS) measurements were performed to further evaluate the potential of the ION-Micelle nanoplatform for MPI. MPS is essentially zero-dimensional MPI and therefore allows to probe the suitability of iron oxide nanoparticles for MPI purposes [26]. In addition to the ION-Micelles, also three commercially available iron oxide formulations (Endorem, Resovist and Sinerem) were measured as a benchmark. MPS measurements were performed using a dedicated magnetic particle spectrometer and each sample was acquired over thirty seconds upon application of an oscillating magnetic field with an amplitude of 10 mT at 25 kHz and RT. ION-Micelles generated increased MPS signals in comparison to the commercially available iron oxide nanoparticles over the entire frequency range (Figure 5C). For the lower frequencies (<0.5 MHz), the ION-Micelles generated 4–6 times more signal per gram iron than Resovist, which was the best performing benchmark formulation with respect to inducing an MPS signal. At higher frequencies (>1 MHz), the improvement in ION-Micelle MPS signal compared to that of the other preparations was even more profound: the ION-Micelles generated an MPS signal per gram iron that was more than a factor 200 higher than that of the commercially available iron oxides. Similar results were obtained for samples that were diluted in whole blood (Figure S2). These findings support the hypothesis that the ION-Micelle nanoplatform will allow significantly more sensitive MPI than Resovist, Endorem and Sinerem.

In addition to MPI, iron oxides are frequently employed for CA-MRI purposes. To evaluate the potential of the ION-Micelle nanoplatform for CA-MRI, proton relaxometry measurements were performed. The ION-Micelles displayed a longitudinal relaxivity r_1_ of 6.7 mM^−1^ s^−1^ and a transversal relaxivity r_2_ of 253 mM^−1^ s^−1^ (Table 1). Both the transversal relaxivity and the ratio of transversal/longitudinal relaxivity of the ION-Micelles were much higher than the corresponding values found for all tested commercially available iron oxide compounds. Thus, the ION-Micelles allow for more sensitive MR imaging than the three benchmark formulations.

**Figure 6:**
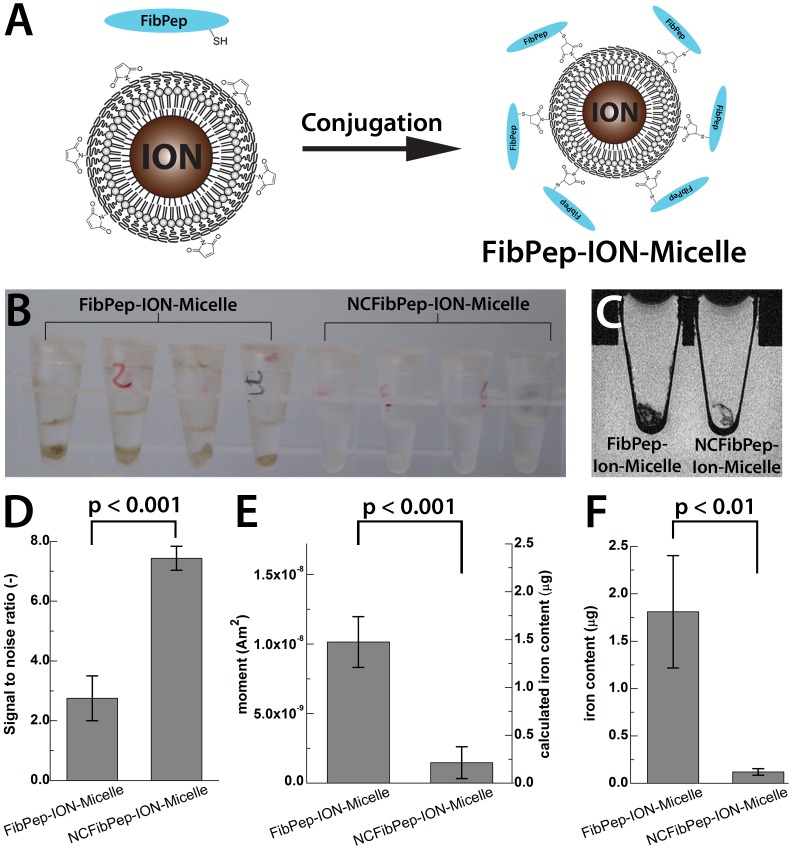
*In vitro* blood clot binding test using FibPep-ION-Micelles and negative control NCFibPep-ION-Micelles. (A) Schematic overview of fibrin-binding peptides (FibPep) conjugation to the ION-Micelles. (B) Photograph and (C) sagittal MR image of blood clots incubated with either FibPep-ION-Micelles (left) or NCFibPep-ION-Micelles (right). (D) MR signal to noise ratio of the (NC)FibPep-ION-Micelles incubated clots. (E) Third harmonic (76 kHz) MPS signal amplitude and estimated iron content of blood clots incubated with (NC)FibPep-ION-Micelles. Estimated iron content was calculated using the third harmonic MPS signal and a previously defined conversion factor for this particular batch IONs of 6.87 mAm^2^/g Fe. (F) Iron content of blood clots incubated with (NC)FibPep-ION-Micelles. Data represents mean ± standard deviation (n = 4).

A proof of concept, *in vitro* blood clot-targeting experiment was performed using fibrin-targeted ION-Micelles to evaluate the suitability of the ION-Micelle nanoplatform for molecular MPI and MRI. Fibrin is a major component of blood clots and plays an important role in thrombi-related pathologies such as deep venous thrombosis, pulmonary embolism and atherosclerosis. Because of the high potency of the ION-Micelle nanoplatform to generate contrast in MPI and MRI, a fibrin-targeted ION-Micelle nanoplatform was envisioned to be suitable for non-invasive detection of thrombi using MPI and/or MRI. To this aim, ION-Micelles were modified with fibrin-binding peptides (FibPeps), which were directly linked to the lipid coating of the nanoparticles. FibPep is a peptide constituted of a fibrin-binding motif (RWQPCPAESWT-Cha-CWDP) [30], [31], which is coupled to an n-succinimidyl-s-acetylthioacetate (SATA) group via a glycine linker (Figure S1A-B). To facilitate conjugation of FibPep to the nanoplatform, the coating of the ION-Micelles was adapted by exchanging 10% of the PEG2000-DSPE lipids with maleimide-functionalized PEG2000-DSPE lipids during the phase-transfer process. Prior to conjugation of the fibrin-binding peptides to the ION-Micelles, the SATA group of the peptides was deacetylated to provide a functional thiol group (Figure S1C). Subsequently, the deacetylated fibrin-binding peptides were conjugated to the ION-Micelles using standard maleimide-thiol chemistry, forming a covalent thioether linkage between the lipidic coating of the nanoparticles and the fibrin-binding peptides (FibPep-ION-Micelles, Figure 6A). As a negative control, a scrambled peptide with C-A substitutions (NCFibPep, Figure S1D–F) was synthesized and coupled to the thiol-modified ION-Micelles, to obtain non-targeting NCFibPep-ION-Micelles.

Blood clots were prepared by incubating a mixture of human tissue factor, calcium chloride and citrated human blood plasma for 30 minutes at 37 °C. Next, blood clots were incubated with HBS containing either FibPep-ION-Micelles or NCFibPep-ION-Micelles for 1 hour (n = 4 per preparation). Subsequently, the solution (containing unbound ION-Micelles) was carefully removed and the clots were washed three times with HBS. Thereafter, clots were subjected to MRI and MPS measurements. A photograph of the clots after the incubation and washing procedure is shown in Figure 6B. The clots incubated with FibPep-ION-Micelles had obtained a brownish color, whereas the clots incubated with the NCFibPep-ION-Micelles had remained white. This is a strong indication that the (brownish-colored) FibPep-ION-Micelles bound specifically to the clots. MRI measurements showed clear signal voids for these clots that were incubated with FibPep-ION-Micelles, whereas those clots that were incubated with NCFibPep-ION-Micelles did not (Figure 6C and Figure S3A). The signal to noise ratio (SNR) of the clots incubated with FibPep-ION-Micelles was significantly lower than the NCFibPep-ION-Micelles incubated clots SNR (2.75 ± 0.75 and 7.43 ± 0.40, respectively; p<0.001; Figure 6D). MPS measurements displayed increased signal amplitudes for the FibPep-ION-Micelles incubated clots in comparison to the NCFibPep-ION-Micelles incubated clots throughout the whole frequency spectrum up to ∼750 kHz (Figure S3B). Above ∼750 kHz, the signal dropped into noise level for both the FibPep-ION-Micelles as well as the NCFibPep-ION-Micelles incubated clots. Specific analysis of the third harmonic (72.6 kHz), which is the harmonic with the highest signal amplitude, showed a seven-fold increase in signal for the FibPep-ION-Micelles incubated clots in comparison to the NCFibPep-ION-Micelles incubated clots (10.1 ± 1.8 and 1.5 ± 1.1 nAm^2^ at 76 kHz, respectively; p<0.001; Figure 6E). The estimated iron content was calculated to be 1.48 ± 0.27 and 0.21 ± 0.17 µg Fe for FibPep-ION-Micelles and NCFibPep-ION-Micelles incubated clots, respectively, using the third harmonic MPS signal and a previously determined conversion factor of 6.87 mAm^2^/g Fe for this particular batch of IONs. Thus, the targeting of the FibPep-ION-Micelles to the clots could be detected using MRI and MPS. To validate whether the FibPep-ION-Micelles indeed bound significantly more to the clots than the NCFibPep-ION-Micelles, the clots were analyzed for iron content using inductively coupled plasma atomic emission spectrometry (ICP-AES). ICP-AES measurements showed that the FibPep-ION-Micelles incubated clots contained significantly more iron than the NCFibPep-ION-Micelles (1.81 ± 0.59 and 0.12 ± 0.03 µg Fe, respectively; p<0.01; Figure 6F). The ICP-AES results match well with the estimated iron content that was calculated from the MPS signal. Hence, these results show that the FibPep-ION-Micelles can selectively bind to blood clots and that this targeting can be visualized using MRI and quantified using MPS. These findings therefore underline the potential of the ION-Micelle nanoplatform for molecular MPI and MRI and encourage future assessment of the FibPep-ION-Micelle nanoplatform for non-invasive detection of thrombi *in vivo* using MRI and MPI.

## Conclusions

Currently, MPI research is mainly being performed employing commercially available iron oxide formulations which were developed for contrast-enhanced MRI. However, these iron oxide formulations contain nanocrystals of which the size is too small and disperse to induce efficiently MPI signal. Therefore, iron oxide formulations optimized for MPI are likely critical in the fruition of MPI as established imaging modality. In this work, we have presented ION-Micelles as a novel nanoplatform, consisting of 25 nm sized iron oxide nanocrystals encapsulated in lipidic micelles, for sensitive application in (molecular) MPI and MRI. The ION-Micelles iron oxide nanocrystals displayed a narrow size-distribution, high saturation magnetization and an effective magnetic core size of 16 nm. In addition, ION-Micelles had a higher potency to generate contrast in MPI and MRI than commercially available iron-oxide formulations (Endorem, Resovist, Sinerem). Thus, ION-Micelles allow for more sensitive MPI and MRI than the currently available iron oxide formulations. Furthermore, the potential of the ION-Micelle platform for molecular MPI and/or MRI was illustrated by an *in vitro* blood clot-targeting experiment using ION-Micelles which were functionalized with fibrin-binding peptides. The fibrin-targeted ION-Micelles (FibPep-ION-Micelles) bound specifically to the blood clots and this could be detected by MPS and MRI. Overall, these findings underline the potential of the ION-Micelle nanoplatform for (molecular) MPI and MRI and warrant further investigation of the FibPep-ION-Micelle platform for *in vivo*, non-invasive imaging of fibrin in preclinical disease models of thrombus-related pathologies and atherosclerosis.

## Supporting Information

Figure S1 **Structural formulas and corresponding mass spectra of FibPep and NCFibPep.** (A,B) FibPep and (D,E) NCFibPep prior to deacetylation. ∼1 hour before conjugation of the peptides to the ION-Micelles, the SATA group is deacetylated in order to obtain a functional thiol group that can bind to the maleimide-functionalized PEG2000-DSPE lipids on the surface of the ION-Micelles (C,F for FibPep and NCFibPep, respectively).
(TIF)

Figure S2 **Magnetic particle spectrometry in whole blood.** MPS experimental data of the ION-Micelles, Endorem, Resovist and Sinerem in whole blood plotted as magnetic moment (normalized for iron content) versus frequency.
(TIF)

Figure S3 **MRI and MPS measurements of blood clots incubated with either FibPep-ION-Micelles or NCFibPep-ION-Micelles.** (A) Transversal MR slice of the blood clots; F  =  clots incubated with FibPep-ION-Micelles; N  =  clots incubated with NCFibPep-ION-Micelles. (B) MPS spectrum of clots incubated with FibPep-ION-Micelles or NCFibPep-ION-Micelles.
(TIF)
