# Supplementary figures and images for: Iron Oxide Nanoparticle-Micelles (ION-Micelles) for Sensitive (Molecular) Magnetic Particle Imaging and Magnetic Resonance Imaging
Source: PLoS One. 2013 Feb 20;8(2):e57335. doi: 10.1371/journal.pone.0057335 (PMC3577714; doi:10.1371/journal.pone.0057335)

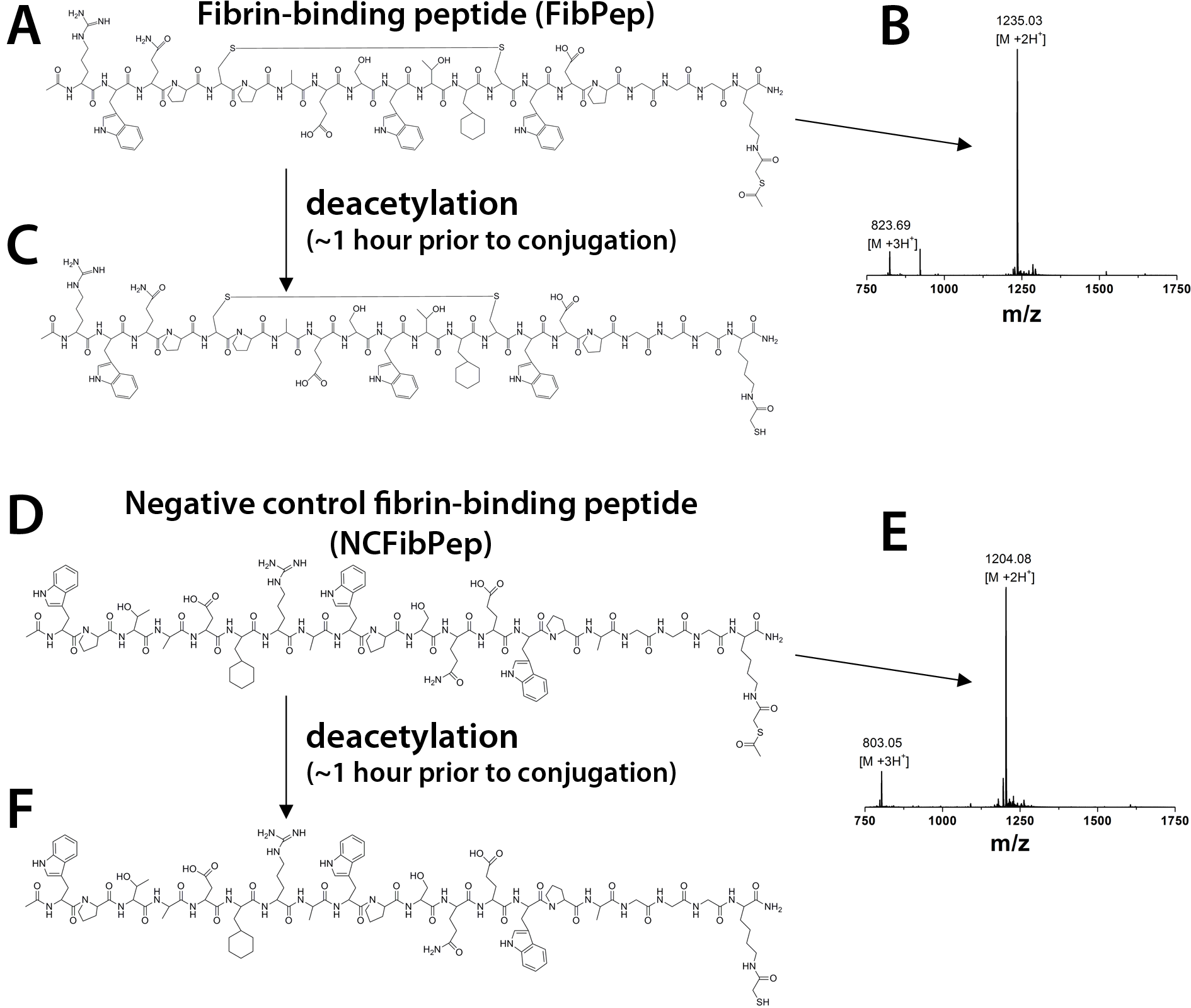

Supplement: Figure S1 — Structural formulas and corresponding mass spectra of FibPep and NCFibPep. (A,B) FibPep and (D,E) NCFibPep prior to deacetylation. ∼1 hour before conjugation of the peptides to the ION-Micelles, the SATA group is deacetylated in order to obtain a functional thiol group that can bind to the maleimide-functionalized PEG2000-DSPE lipids on the surface of the ION-Micelles (C,F for FibPep and NCFibPep, respectively). (TIF) [file pone.0057335.s001.tif]

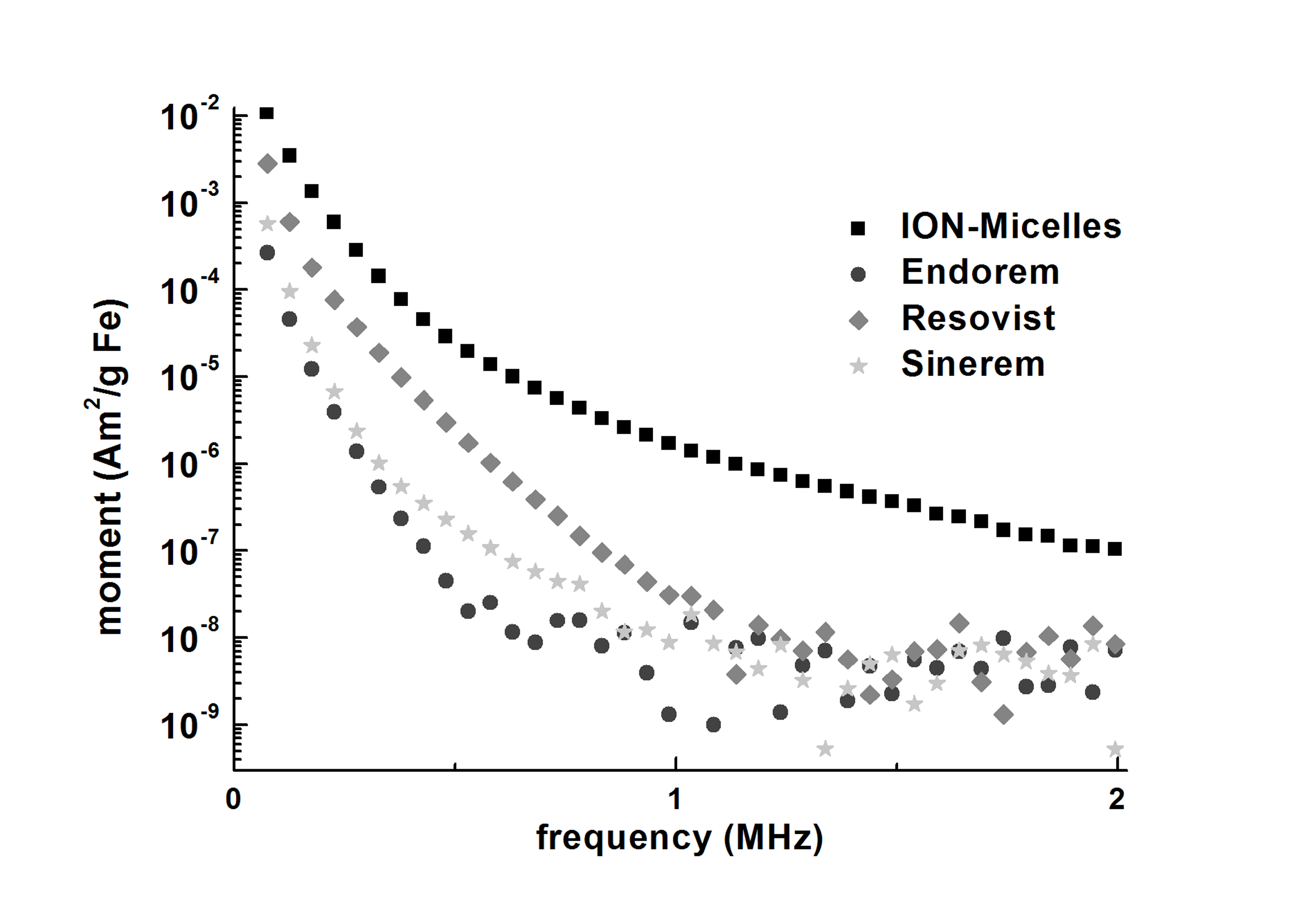

Supplement: Figure S2 — Magnetic particle spectrometry in whole blood. MPS experimental data of the ION-Micelles, Endorem, Resovist and Sinerem in whole blood plotted as magnetic moment (normalized for iron content) versus frequency. (TIF) [file pone.0057335.s002.tif]

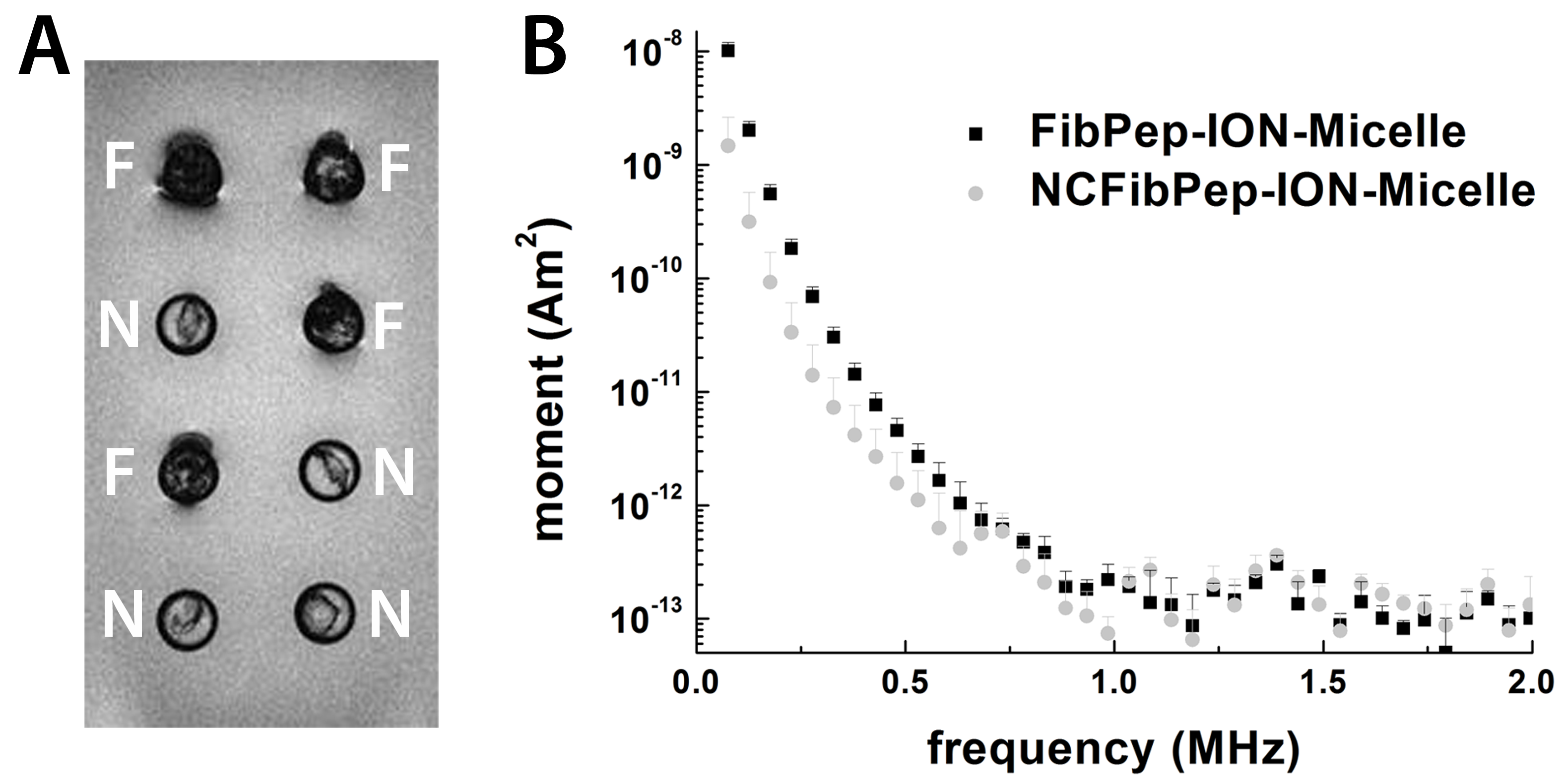

Supplement: Figure S3 — MRI and MPS measurements of blood clots incubated with either FibPep-ION-Micelles or NCFibPep-ION-Micelles. (A) Transversal MR slice of the blood clots; F = clots incubated with FibPep-ION-Micelles; N = clots incubated with NCFibPep-ION-Micelles. (B) MPS spectrum of clots incubated with FibPep-ION-Micelles or NCFibPep-ION-Micelles. (TIF) [file pone.0057335.s003.tif]
